# Supplementary material for: Bifurcation drives the evolution of assembly-line biosynthesis
Source: Nat Commun. 2022 Jun 17;13:3498. doi: 10.1038/s41467-022-30950-z (PMC9205934; doi:10.1038/s41467-022-30950-z)
Supplement: Supplementary file 1 — Supplementary Information [file 41467_2022_30950_MOESM1_ESM.pdf]

Supplementary Information

Bifurcation Drives the Evolution of Assembly-Line Biosynthesis

Thomas J. Booth, Kenan A. J. Bozhüyük, Jonathon D. Liston, Sibyl F. D. Batey, Ernest Lacey & Barrie Wilkinson*

Contents

1. Supplementary Figures..2
2. Supplementary Tables..16
3. Supplementary References..31

1. Supplementary Figures

1.1 Supplementary Figure 1: Cluster Maps of Hexapeptide producing BGCs

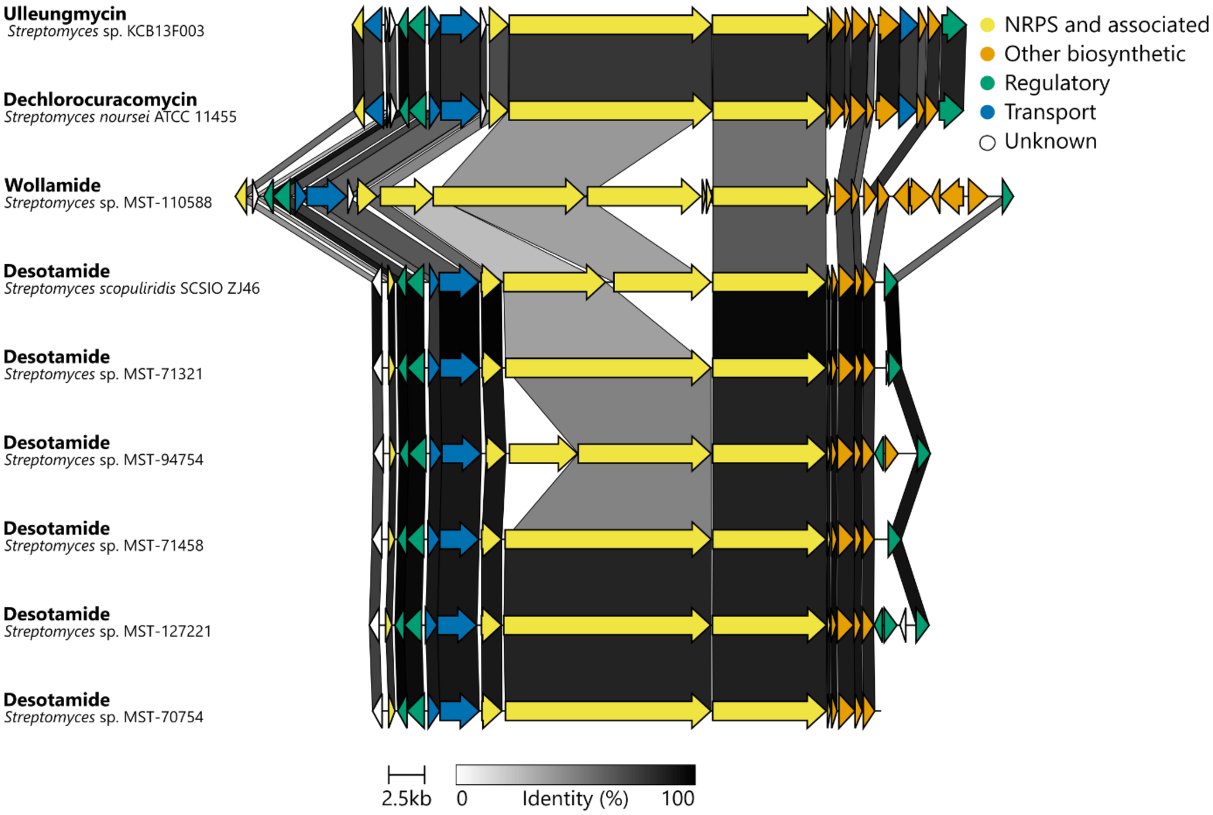

Comparison of hexapeptide producing strains from the Microbial Screening Technologies collection (MST-110588, MST-71321, MST-94754, MST-127221 and MST-70754 (this study)) and previously published hexapeptide BGCs for desotamide[1], dechlorocuracomycin[2] and ulleungmycin[3]. Figure adapted from clinker output[4].

1.2 Supplementary Figure 2: Plasmid Map of pBO1.

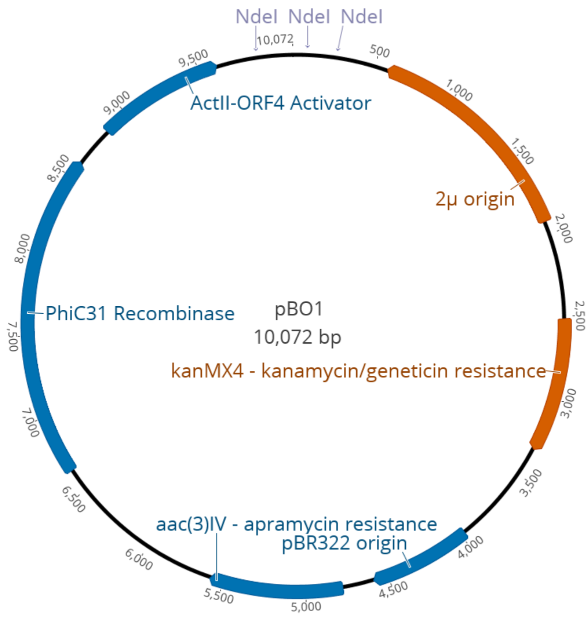

Regions derived from pGP9[5] are coloured blue; those derived from pFF62A[6] are coloured red.

1.3 Supplementary Figure 3: TICs and EICs of desotamide A and wollamide A standards and producing strains.

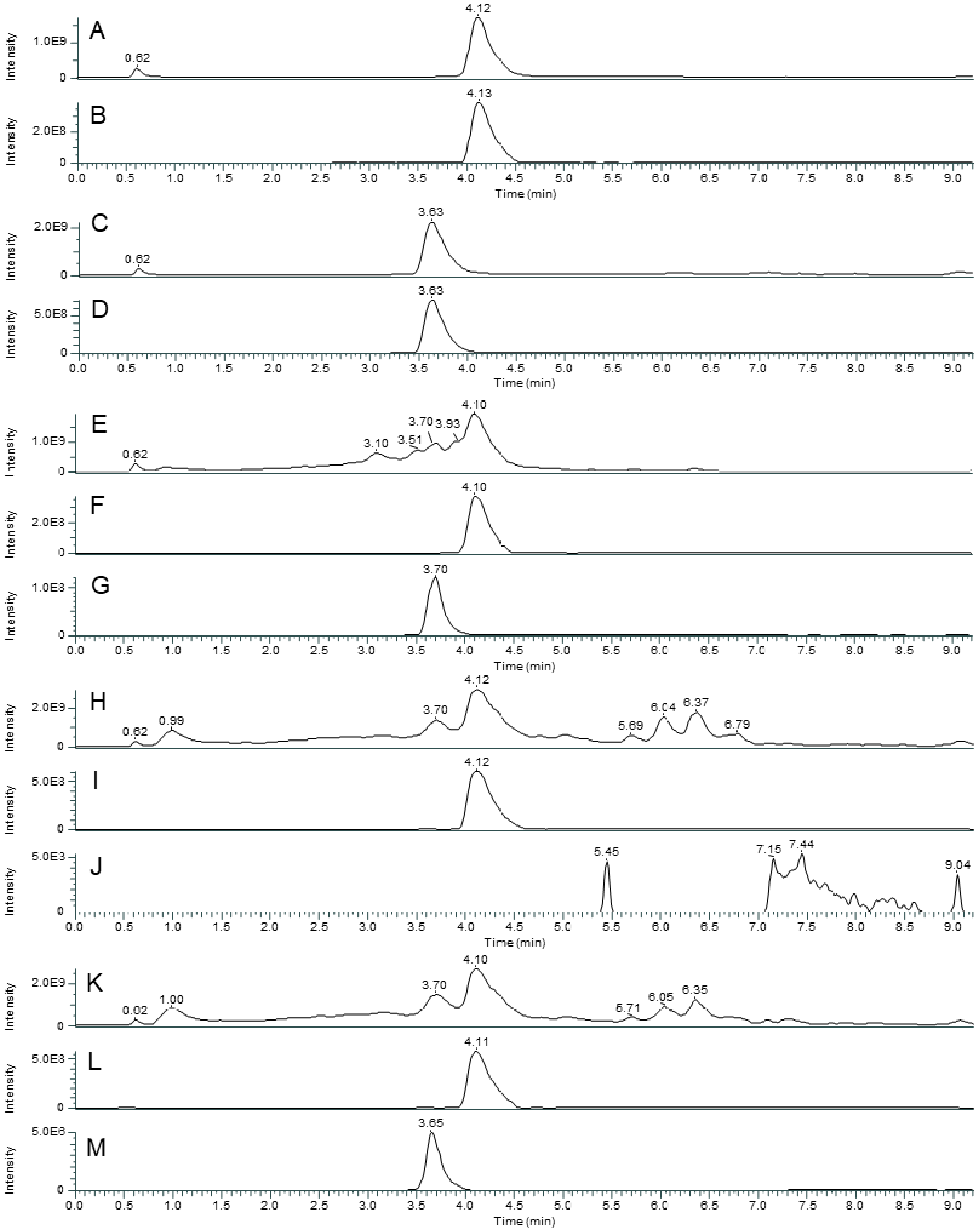

Total ion chromatograms (TIC) and extracted ion chromatograms (EIC) showing the following: A: TIC of desotamide A standard; B: EIC of desotamide A standard ($[M+H]^+$ = 697.40); C: TIC of wollamide A standard; D: EIC of wollamide A standard ($[M+H]^+$ = 754.46); E: TIC from 110588 extract; F: EIC of desotamide A (697.40) from 110588 extract; G: EIC of wollamide A (754.46) in 110588 extract; H: TIC of 70754 extract; I: EIC of desotamide A (697.40) from 70754 extract; J: EIC of wollamide A (754.46) from 70754 extract (noise); K: TIC of 70754/pBO1-*wolG2* extract; I: EIC of desotamide A (697.40) from 70754/pBO1-*wolG2* extract; J: EIC of wollamide A (754.46) from 70754/pBO1-*wolG2* extract.

1.4 Supplementary Figure 4: Average mass spectra for desotamide A and wollamide A peaks

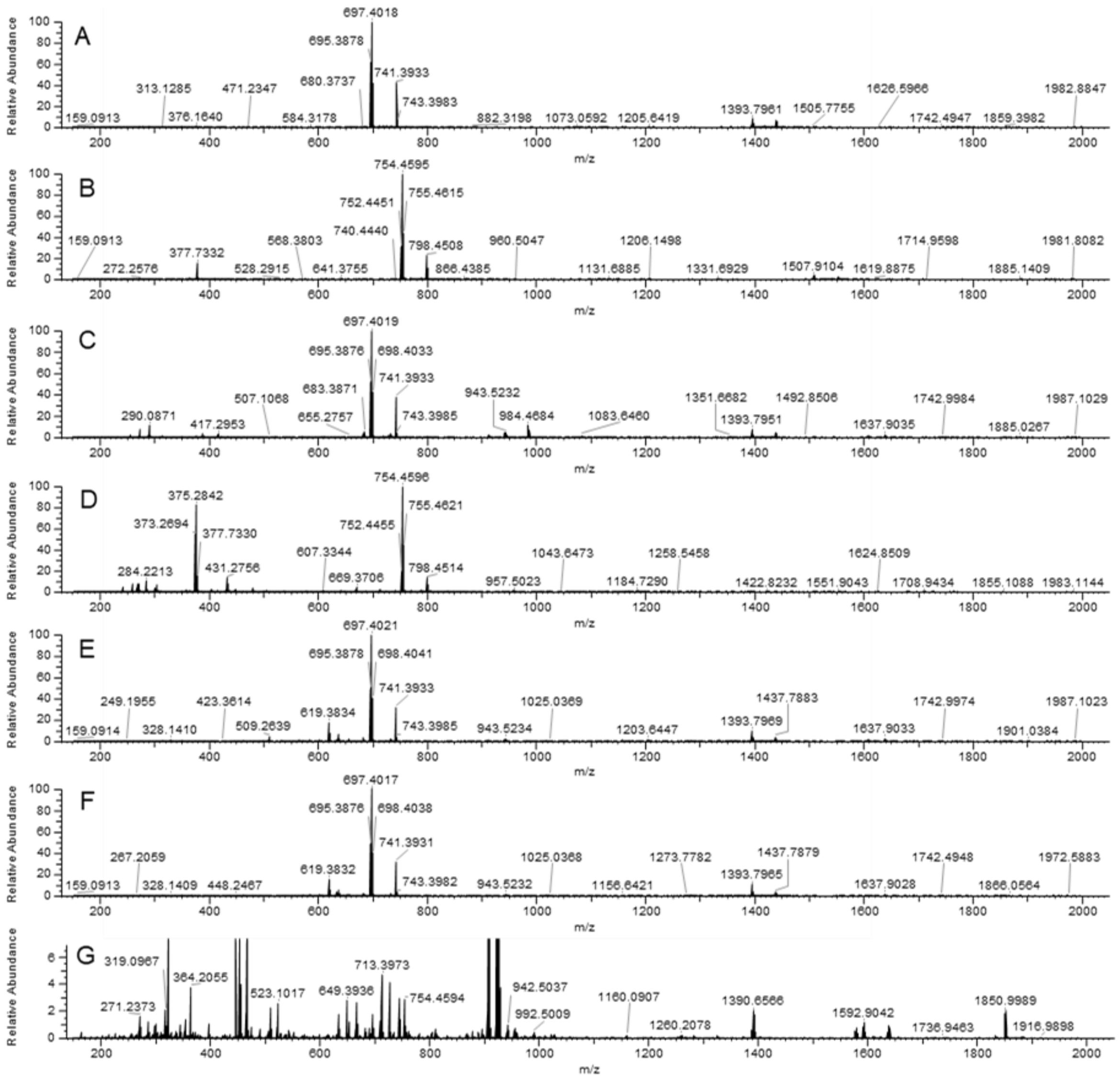

Mass spectra averaged across the peaks in Supplementary Figure 3. A: desotamide A standard; B: wollamide A standard; C: 110588 desotamide A; D: 110588 wollamide A; E: 70754 desotamide A; F: 70754/pBO1-wolG2 desotamide A; G: 70754/pBO1-*wolG2* wollamide A. Note: Spectra G is amplified due to overlapping peaks.

1.5 Supplementary Figure 5: MS/MS fragmentation of desotamide A and wollamide A

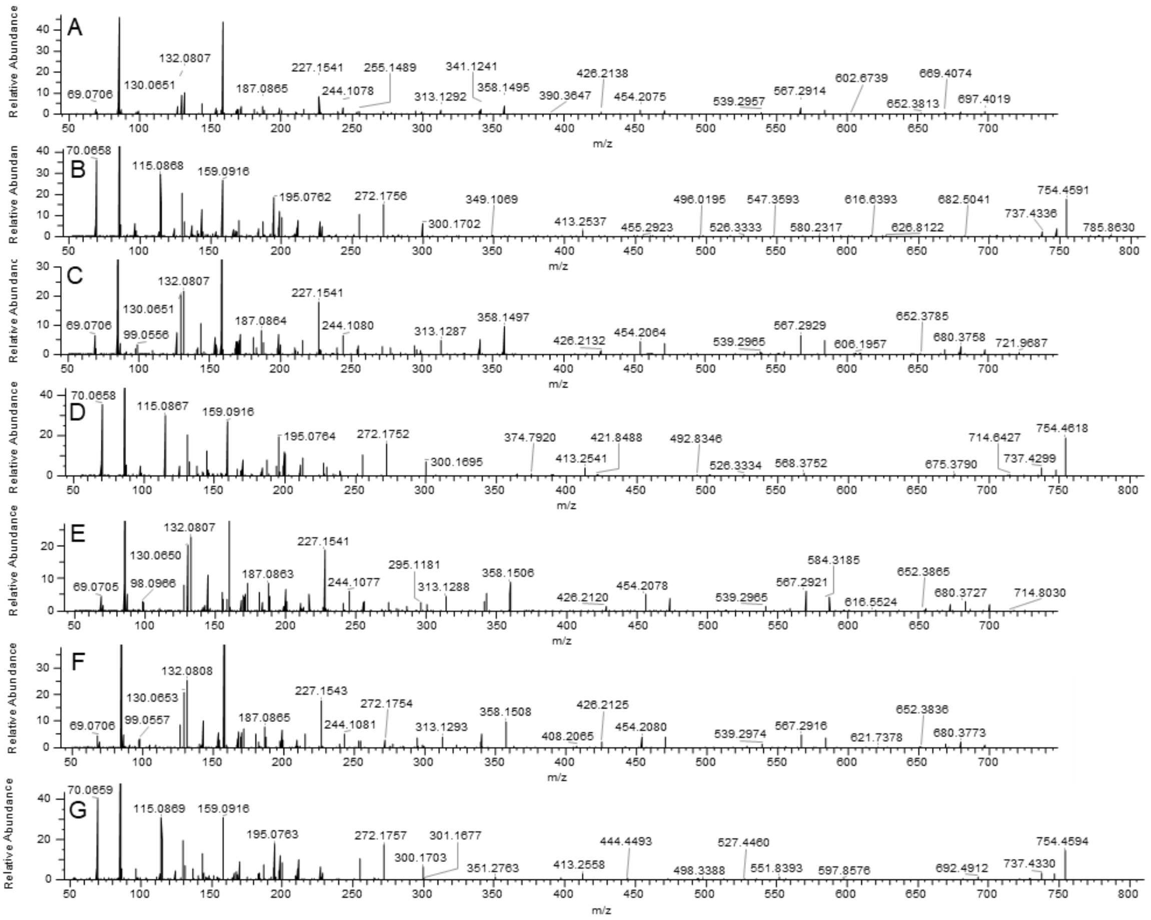

MS/MS fragmentation of desotamide A and wollamide A. A: desotamide A standard; B: wollamide A standard; C: desotamide A from 110588 extracts; D: wollamide A from 110588 extracts; E: desotamide A from 70754 extracts; F: desotamide A from 70754/pBO1-*wolG2;* G: wollamide A from 70754/pBO1-*wolG2.*

1.6 Supplementary Figure 6: Sequences of docking-domain complexes used for homology modeling.

6TRP_1|Chain

MNINEQTLDKLRQAVLQKKIKERIQNSLSTEKY **GSGSGSGSGSGSGSGSGSGSGSGSGY** QIETFFAQDIESVQKELENLSEEELLAMLNGDQQ 93

WolG1 NDD – linker - WolH CDD

MDAAAARESWIQSLP**E**HVRDRMRSRLTGGAQDG **GSGSGSGSGSGSGSGSGSGSGSGSGY** EIFMIDLPA**R**TLFDNPTVADLAAAVEAQVLAELS 93

WolG2 NDD -linker - WolH CDD

MDAAASRESWIQSLP**A**HVRDRMRSRLAGGAEAG **GSGSGSGSGSGSGSGSGSGSGSGSGY** EIFMIDLPA**R**TLFDNPTVADLAAAVEAQVLAELS 93

Specific interactions between different proteins in NRPS systems are mediated by short C- and N-terminal docking domains ($^{C/N}$DDs). The solution structure 6TRP_1 (PaxC_NDD–PaxB_CDD) was used as a template[7] to model both WolG1_NDD–WolH_CDD and WolG2_NDD–WolH_CDD. As artificial linking of the PaxB/PaxC DD pair via a flexible glycine-serine (GS) linker led to the elucidation of the structure of the DD complex by NMR spectroscopy, we applied an analogous in silico approach for homology modeling. Left: N-terminal DD; Center: GS linker; Right: C-terminal DD. Proposed key interaction of the WolG1 WolH DD pair (red) that is missing in the WolG2 WolH pair (blue) is highlighted (c.f. Supplementary Figure S7).

1.7 Supplementary Figure 7: Homology models of WolH/WolG docking-domain complexes.

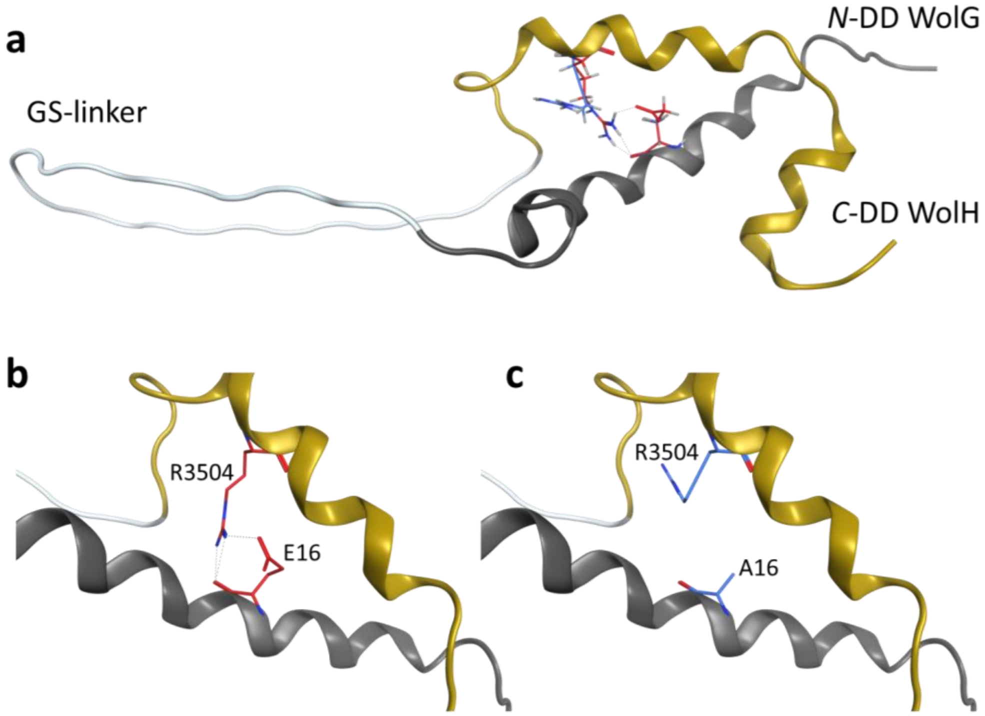

Homology models were calculated to reveal the reason for observed differences in desotamide and wollamide peptide titers. The WolG1 and WolG2 DDs are colored grey, the WolH DDs are colored yellow, and the artificial GS linker are colored white. Depicted amino acid residues (stick representation) are colored as introduced in Supplementary Figure S6. (a) Superimposed models of WolG1 WolH and WolG2 WolH DD pairs have an RMSD of 0.051. (b) Excised WolG1 WolH DD pair. Amino acid residues E16 (WolH) and R3504 (WolG1) are forming a salt bridge. (c) Excised WolG2 WolH DD pair. The E16A amino acid change in WolG2 likely causes decreased DD pair affinities. This in turn may serve as an explanation for lower wollamide titers compared to desotamide titers.

1.8 Supplementary Figure 8: Pairwise identity and comparative GC-Skew of WolG1 and WolG2

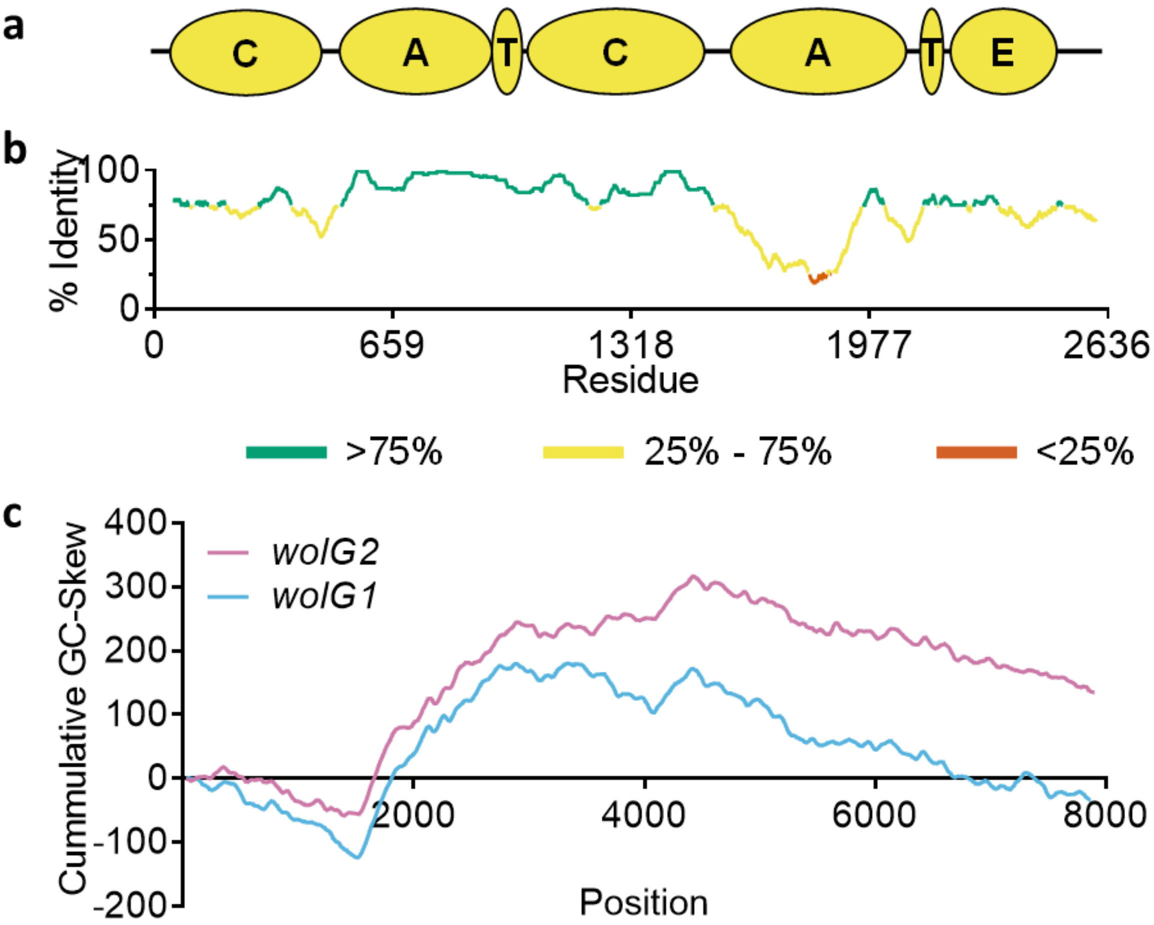

a) The domain architecture of WolG1 is shown across the top of the figure with the domains denoted as follows: condensation (C), adenylation (A), thiolation/peptidyl carrier protein (T) and, epimerase (E). b) The pairwise amino acid identity of WolG1 and WolG2 calculated as a 50-residue sliding window, colour coded as follows: regions of high homology (>75%) are coloured in green, medium homology in yellow (25 -75%) and low homology in red (<25%). c) The cumulative GC-skew measured across *wolG1* and *wolG2.*

1.9 Supplementary Figure 9: Phylogeny of *Streptomyces* sp. MST-110588 Condensation domains

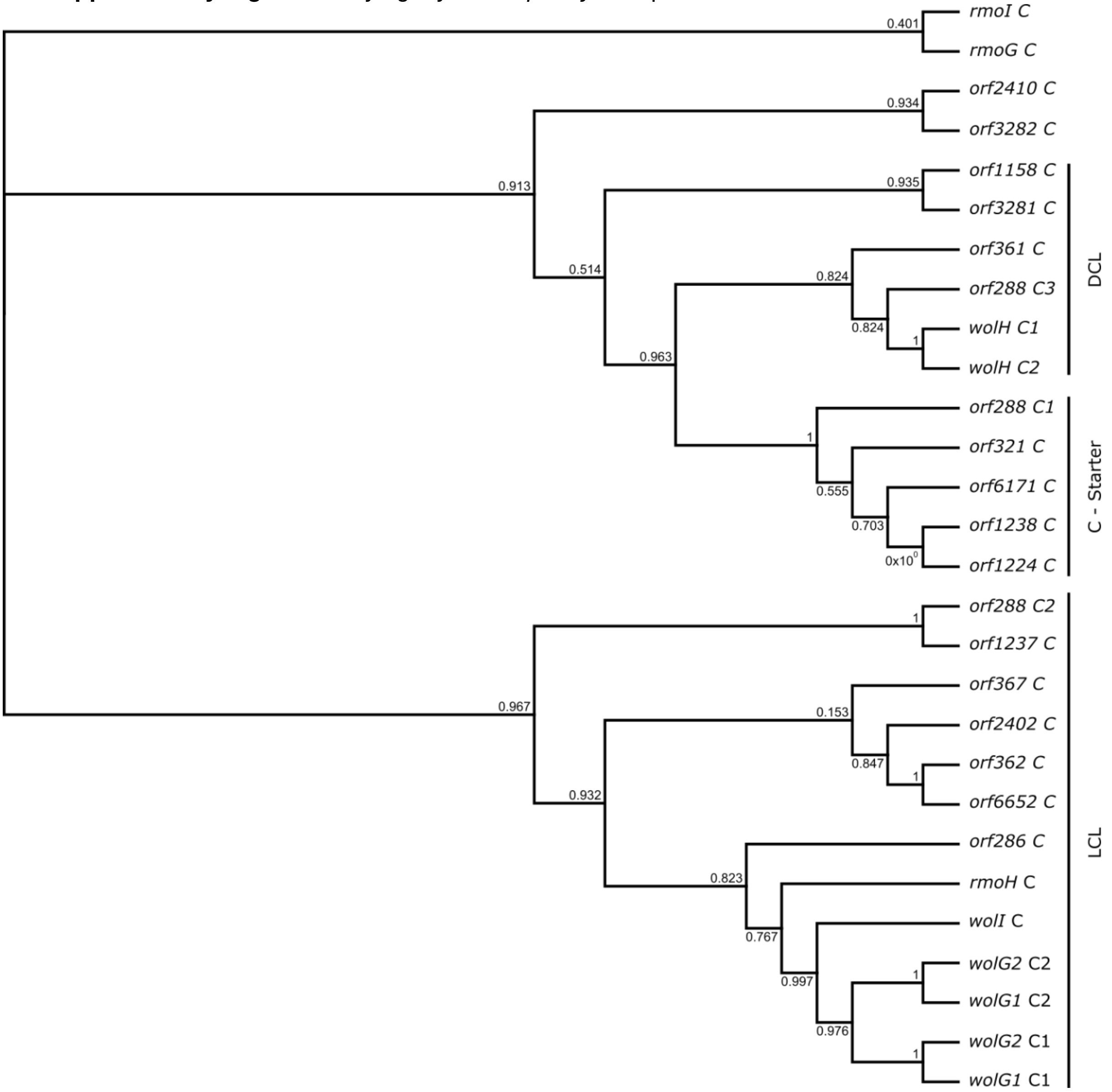

Phylogenetic tree of MST-110588 condensation domains generated by maximum likelihood as implemented in FastTree2[8] from a ClustalW[9] alignment of nucleotide sequences. Condensation domain functions were predicted by NaPDoS[10] major clades are indicated ($^{L}C_{L}$s, which condense two L-amino acid residues; $^{D}C_{L}$s that condense a D-amino acid residues with L-amino acid residues and; C Starter domains, associated with the loading modules of NRPSs).

1.10 Supplementary Figure 10: Phylogeny of *Streptomyces* spp. Adenylation domains (recombinant region)

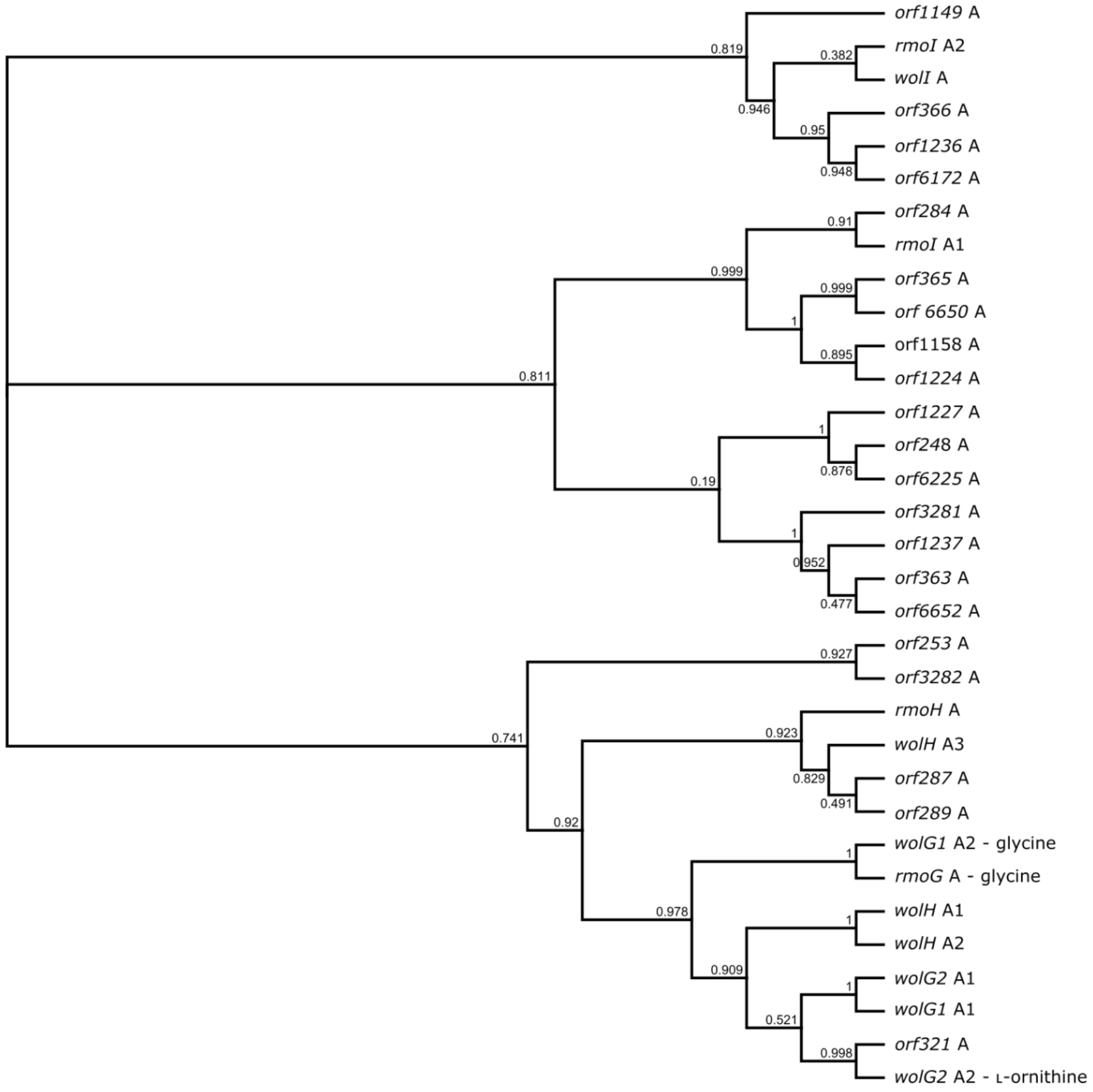

Phylogenetic tree of the *wolG1*/*wolG2/RmoG* recombinant region of MST-110588 adenylation domains (as predicted by RDP4) generated by maximum likelihood as implemented in FastTree2[8] from a ClustalW[9] alignment of nucleotide sequences. Observed substrate specificities encoded by *wolG1*, *wolG2* and *rmoG* are indicated.

1.11 Supplementary Figure 11: Phylogeny of *Streptomyces* spp. Adenylation domains (parental region)

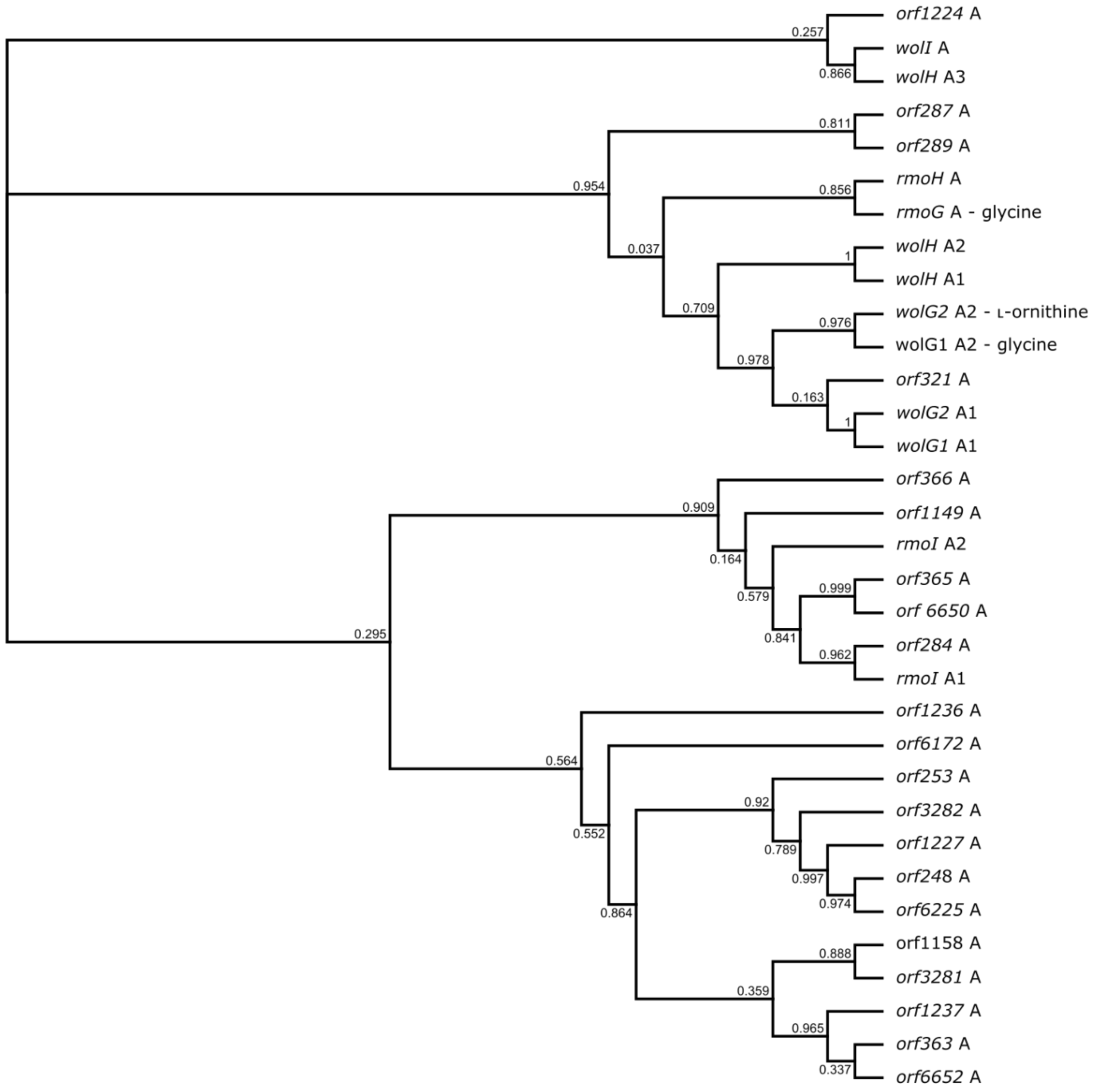

Phylogenetic tree of the of MST-110588 adenylation domains with the *wolG1/wolG2/rmoG* recombinant region removed, generated by maximum likelihood as implemented in FastTree2[8] from a ClustalW[9] alignment of nucleotide sequences. Observed substrate specificities encoded by *wolG1*, *wolG2* and *rmoG* are indicated.

1.12 Supplementary Figure 12: Topology of a typical adenylation domain showing predicted recombination breakpoints

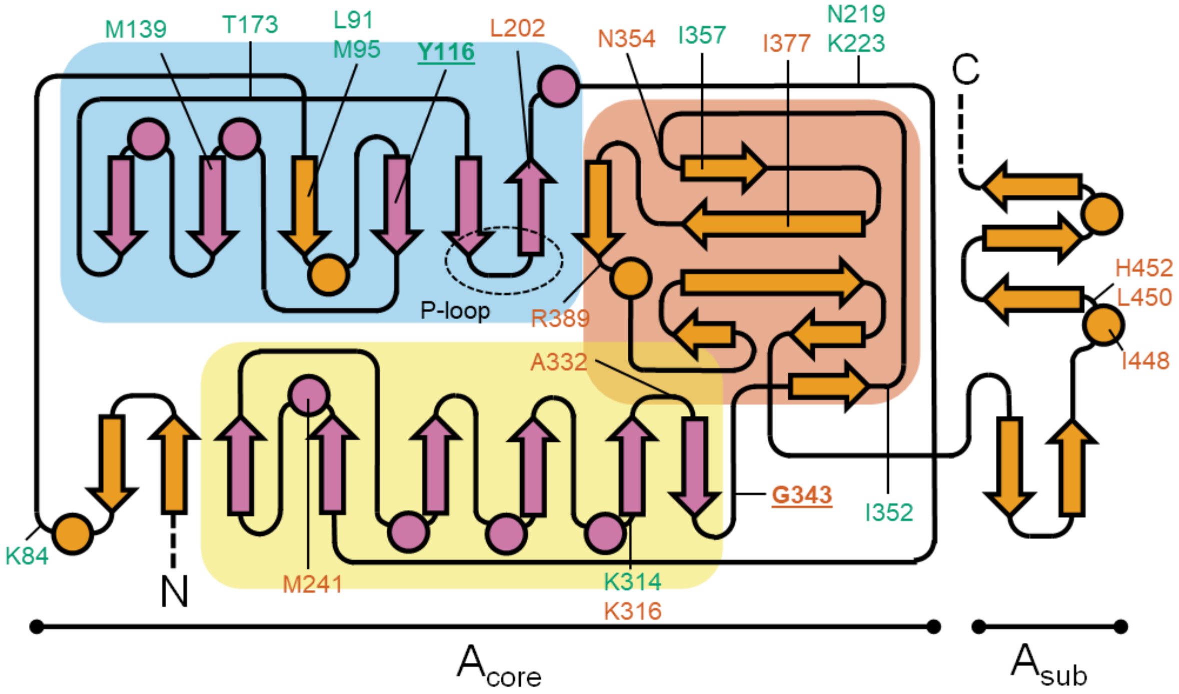

Topology of a typical adenylation domain as reported by Conti et al.[11]. Circles represent α-helices, arrows represent β-strands, and lines represent loops. Regions corresponding to the large *N*-terminal domain (A_{core}) and small *C*-terminal domain (A_{sub}) are highlighted and subdomains shaded (subdomain 1 = blue; subdomain 2 (flavodoxin-like subdomain) = yellow; subdomain 3 = red). The residues indicated correspond to approximate sites of recombination from Supplementary Table 9 (green = N-terminal; red = C-terminal). Recombinant region of *wolG1* coloured in purple and associated breakpoints are underlined.

1.13 Supplementary Figure 13: Homology model of the final module of WolG1

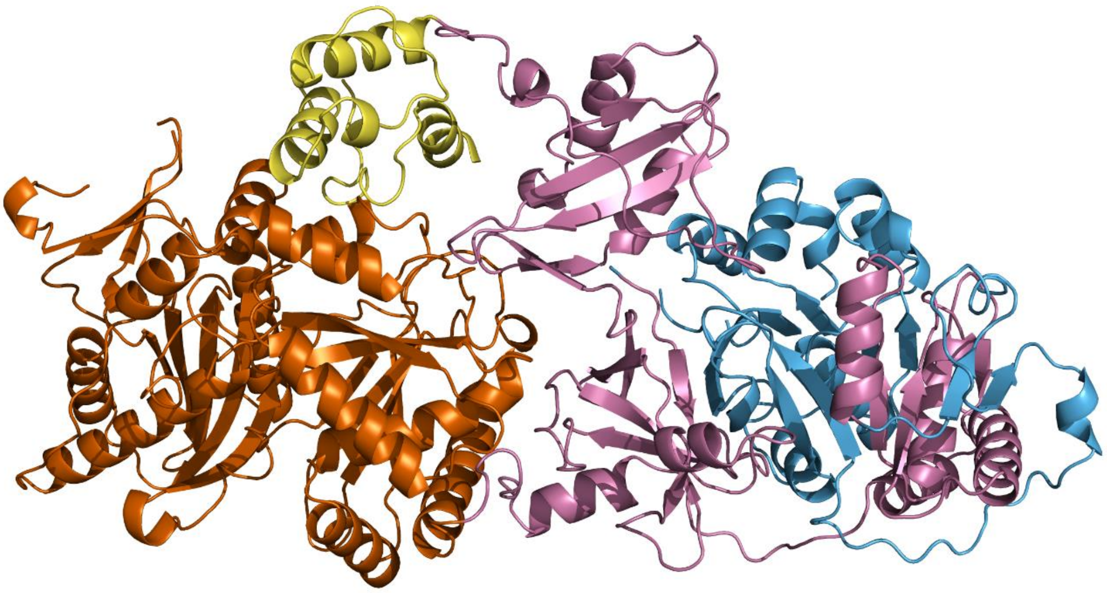

Homology model of WolG1 made with Phyre2[12] based on the structure of surfactin synthetase (PDB: 2VSQ)[13]. The condensation and thiolation domains are coloured orange and yellow, respectively. The adenylation is coloured pink, with the recombinant region coloured in blue.

1.14 Supplementary Figure 14: The evolutionary relationships of the desotamides, wollamides and rimosamides.

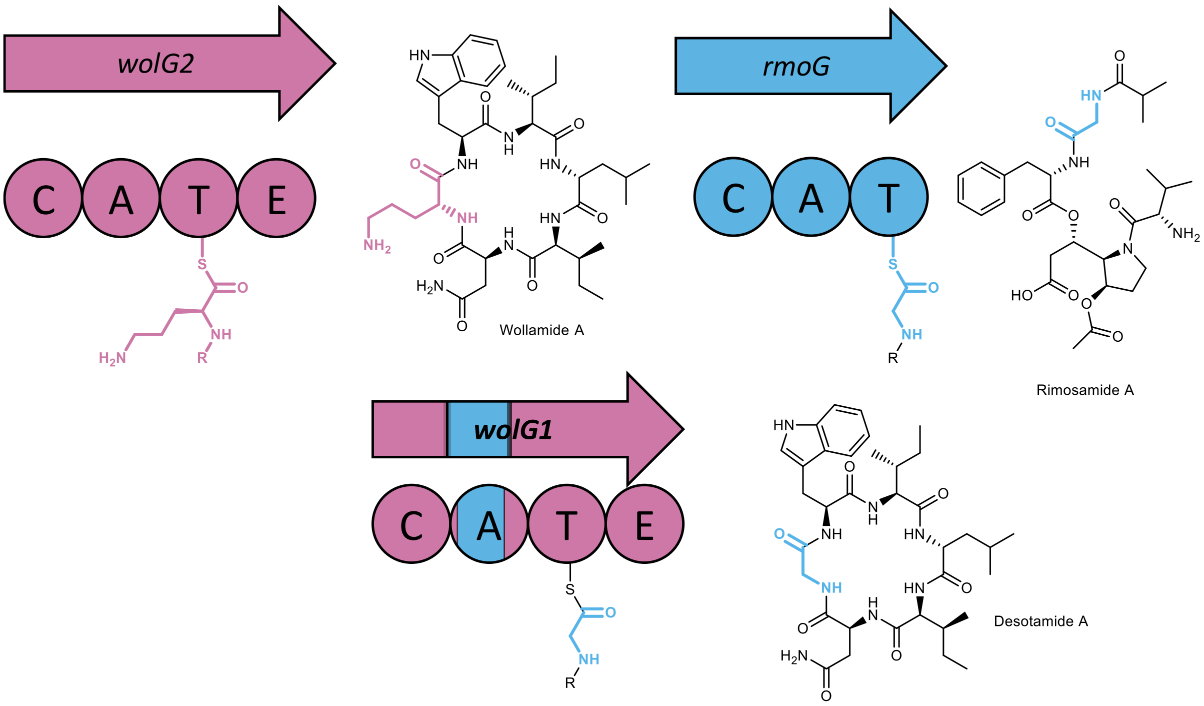

The condensation (C), adenylation (A), thiolation (T) and epimerization (E) domain architecture of the final modules of the WolG1/WolG2 NRPS assembly line enzymes are shown (R corresponds to the proceeding residues of the NRPS bound peptides). Wollamide NRPS shown in pink, rimosamide NRPS shown in blue.

2. Supplementary Tables

2.1 Supplementary Table 1: Genome assembly information for Microbial Screening Technology (MST) *Streptomyces* spp. strains sequenced in this study

	MST-7054	MST-71321	MST-71458	MST-94754	MST-110588	MST-127221
**Sequencing Platform**	Illumina	Illumina	Illumina	Illumina	PacBio	Illumina
**Total Contigs**	4	21	4	6	1	5
**Mean Contig Length**	1,801,242	453,570	2,322,188	1,486,346	7,915,503	1,780,776
**N50**	9,114,431	1,425,667	9,288,751	6,331,616	7,915,503	8,773,046
**Total Bases**	9,114,431	9,524,967	9,288,751	8,918,075	7,915,503	8,903,878

2.2 Supplementary Table 2: BLAST analysis of wollamide (*wol*) BGC from *Streptomyces* sp. MST-110588

Name	Start Position	End Position	Residues	Direction	Top Hit	Identity (%)	Coverage (%)	Role
Orf-1	4,622,717	4,623,274	345	reverse	-	-	-	-
Orf-2	4,623,231	4,624,019	186	forward	ABC transporter [*Streptomyces* sp. KCB13F003]	63	95	Transport
WolP	4,624,440	4,624,853	263	reverse	thioesterase [*Streptomyces* sp. 769]	69	90	Thioesterase
WolO	4,625,211	4,625,879	138	forward	hypothetical protein [*Streptomyces* sp. L-9-10]	52	96	Unknown
WolN	4,625,867	4,627,078	223	reverse	LuxR family transcriptional regulator [*Streptomyces* sp. KCB13F003]	95	99	Regulator
WolM	4,627,420	4,628,238	404	reverse	sensor histidine kinase [*Streptomyces* sp. L-9-10]	82	99	Regulator
WolL	4,628,235	4,631,006	273	forward	putative ABC transport system ATP-binding protein [*Streptomyces yunnanensis*]	79	99	Transport
WolK	4,631,092	4,631,511	924	forward	ABC transporter permease [*Streptomyces* sp. KCB13F003]	71	98	Transport
WolX	4,631,762	4,633,141	140	forward	hypothetical protein [*Streptomyces* sp. KCB13F003]	59	94	Unknown
WolJ	4,633,374	4,637,105	460	forward	DsaJ [*Streptomyces scopuliridis*]	68	99	Peptide Cyclase
WolI	4,637,078	4,647,664	1244	forward	amino acid adenylation domain-containing protein [*Streptomyces* sp. L-9-10]	71	91	NRPS
WolH	4,647,843	4,655,768	3529	forward	NRPS [*Streptomyces* sp. KCB13F003]	67	99	NRPS
WolG2	4,655,877	4,656,086	2642	forward	DsaG [*Streptomyces scopuliridis*]	66	99	NRPS
WolF2	4,656,171	4,656,551	70	forward	MbtH protein [*Streptomyces* sp. KCB13F003]	87	98	NRPS
WolE	4,656,623	4,664,527	127	forward	nuclear transport factor 2 family protein [*Streptomyces* sp. L-9-10]	79	99	Amino acid biosynthesis
WolG1	4,664,584	4,664,835	2635	forward	DsaG [*Streptomyces scopuliridis*]	72	99	NRPS
WolF1	4,665,154	4,666,323	84	forward	MbtH protein [*Streptomyces* sp. KCB13F003]	61	76	NRPS
WolD	4,666,320	4,666,823	390	forward	DsaD [*Streptomyces scopuliridis*]	82	94	Amino acid biosynthesis
WolC	4,667,176	4,668,030	168	forward	YbaK/prolyl-tRNA synthetase associated domain-containing protein [*Streptomyces* sp. L-9-10]	84	98	Unknown
WolW	4,668,143	4,668,934	285	forward	amidinotransferase [*Streptomyces* sp. KCB13F003]	91	93	Amino acid biosynthesis
WolB	4,669,242	4,670,300	264	forward	indole-3-glycerol phosphate synthase TrpC [*Streptomyces* sp. NRRL S-1813]	76	99	Amino acid biosynthesis
WolV	4,622,717	4,623,274	353	reverse	anthranilate phosphoribosyltransferase [*Streptomyces decoyicus*]	83	99	Amino acid biosynthesis

**Name**	**Start Position**	**End Position**	**Residues**	**Direction**	**Top Hit**	**Identity (%)**	**Coverage (%)**	**Role**
WolU	4,670,408	4,671,829	474	forward	PLP-dependent aminotransferase family protein [*Streptomyces decoyicus*]	82	99	Amino acid biosynthesis
WolT	4,671,909	4,672,484	192	reverse	aminodeoxychorismate/anthranilate synthase component II [*Streptomyces decoyicus*]	85	97	Amino acid biosynthesis
WolS	4,672,481	4,674,127	549	reverse	anthranilate synthase component I family protein [*Streptomyces* sp. NRRL S-1813]	69	99	Amino acid biosynthesis
WolR	4,674,491	4,675,834	448	forward	phospho-2-dehydro-3-deoxyheptonate aldolase [*Streptomyces* sp. KCB13F003]	85	97	Amino acid biosynthesis
WolA	4,676,859	4,677,629	257	forward	DsaA [*Streptomyces scopuliridis*]	76	99	Regulator
Orf+1	4,678,250	4,678,567	106	reverse	DUF4190 domain-containing protein [*Streptomyces varsoviensis*]	70	79	-
Orf+2	4,678,564	4,678,728	55	reverse	hypothetical protein [*Streptomyces albospinus*]	74	98	-
Orf+3	4,678,852	4,680,309	486	reverse	M6 family metalloprotease domain-containing protein [Streptomyces sp. ID38640]	65	79	-

Supplementary Table 2 continued.

2.3 Supplementary Table 3: BLAST analysis of desotamide (*dsa*) BGC from *Streptomyces* sp. MST-70754

Name	Start Position	End Position	Residues	Direction	Top Hit	Identity (%)	Coverage (%)
Orf-6	1	984	328	forward	hypothetical protein [*Streptomyces scopuliridis*]	89	99
Orf-5	1113	3332	740	reverse	NADP-dependent isocitrate dehydrogenase [*Streptomyces* sp. L-9-10]	98	99
Orf-4	3435	3821	129	reverse	D-ribose pyranase [*Streptomyces scopuliridis*]	93	99
Orf-3	3818	4798	327	reverse	ribokinase [*Streptomyces scopuliridis*]	89	99
Orf-2	4848	6824	659	reverse	inner-membrane translocator [*Streptomyces scopuliridis*]	99	99
Orf-1	6814	8370	519	reverse	ATP-binding protein [*Streptomyces scopuliridis*]	99	99
DsaQ	8367	9506	380	reverse	DsaQ [*Streptomyces scopuliridis*]	98	99
DsaX	9997	10614	206	forward	hypothetical protein [*Streptomyces* sp. L-9-10]	77	77
DsaP	10517	11188	224	reverse	DsaP [*Streptomyces scopuliridis*]	97	99
DsaO	11642	12091	150	forward	DsaO [*Streptomyces scopuliridis*]	98	99
DsaN	12264	12932	223	reverse	response regulator transcription factor [*Streptomyces* sp. L-9-10]	98	99
DsaM	12920	14131	404	reverse	DsaM [*Streptomyces scopuliridis*]	98	99
DsaL	14411	15229	273	forward	ABC transporter ATP-binding protein [*Streptomyces* sp. L-9-10]	93	99
DsaK	15235	17994	920	forward	DsaK [*Streptomyces scopuliridis*]	99	99
DsaJ	18164	19573	470	forward	DsaJ [*Streptomyces scopuliridis*]	99	99
DsaH	19805	34177	4791	forward	NRPS [*Streptomyces* sp. KCB13F003]	65	99
DsaG	34298	42241	2648	forward	DsaG [*Streptomyces scopuliridis*]	97	99
DsaF	42320	42529	70	forward	DsaF [*Streptomyces scopuliridis*]	100	98
DsaE	42618	42992	125	forward	DsaE [*Streptomyces scopuliridis*]	98	99
DsaD	43122	44255	378	forward	DsaD [*Streptomyces scopuliridis*]	98	99
DsaC	44252	44755	168	forward	DsaC [*Streptomyces scopuliridis*]	99	99
DsaB	44826	45638	271	forward	DsaB [*Streptomyces scopuliridis*]	98	99
DsaY	46439	46624	62	forward	-	-	-
DsaA	46599	47516	306	forward	DsaA [*Streptomyces scopuliridis*]	99	99
Orf+1	47650	48351	234	reverse	hydrolase [*Streptomyces scopuliridis*]	98	99
Orf+2	48365	48847	161	reverse	transcriptional regulator [*Streptomyces scopuliridis*]	100	99
Orf+3	48905	50305	467	reverse	hypothetical protein [*Streptomyces scopuliridis*]	89	99

2.4 Supplementary Table 4: BLAST analysis of desotamide (*dsa*) BGC from *Streptomyces* sp. MST-71321

**Name**	**Start Position**	**End Position**	**Residues**	**Direction**	**Top Hit**	**Identity (%)**	**Coverage (%)**
Orf-1	1	618	206	forward	hypothetical protein [*Streptomyces* sp. L-9-10]	77	77
DsaP	521	1192	224	reverse	DsaP [*Streptomyces scopuliridis*]	97	99
DsaO	1655	2104	150	forward	DsaO [*Streptomyces scopuliridis*]	97	99
DsaN	2277	2945	223	reverse	response regulator transcription factor [*Streptomyces* sp. L-9-10]	98	99
DsaM	2933	4144	404	reverse	DsaM [*Streptomyces scopuliridis*]	99	99
DsaL	4424	5242	273	forward	ABC transporter ATP-binding protein [*Streptomyces* sp. L-9-10]	93	99
DsaK	5248	8007	920	forward	DsaK [*Streptomyces scopuliridis*]	99	99
DsaJ	8177	9586	470	forward	DsaJ [*Streptomyces scopuliridis*]	99	99
DsaH	9818	24172	4785	forward	NRPS [*Streptomyces* sp. KCB13F003]	65	99
DsaG	24296	32239	2648	forward	DsaG [*Streptomyces scopuliridis*]	97	99
DsaF	32324	32533	70	forward	DsaF [*Streptomyces scopuliridis*]	100	98
DsaE	32603	32977	125	forward	DsaE [*Streptomyces scopuliridis*]	98	99
DsaD	33110	34243	378	forward	DsaD [*Streptomyces scopuliridis*]	98	99
DsaC	34240	34743	168	forward	DsaC [*Streptomyces scopuliridis*]	99	99
DsaB	34813	35625	271	forward	DsaB [*Streptomyces scopuliridis*]	97	99
DsaY	36438	36623	62	forward	-	-	-
DsaA	36598	37515	306	forward	DsaA [*Streptomyces scopuliridis*]	99	99
Orf+1	37641	38342	234	reverse	hydrolase [*Streptomyces scopuliridis*]	98	99
Orf+2	38356	38838	161	reverse	transcriptional regulator [*Streptomyces scopuliridis*]	100	99
Orf+3	38896	40293	466	reverse	hypothetical protein [*Streptomyces scopuliridis*]	90	99

2.5 Supplementary Table 5: BLAST analysis of desotamide (*dsa*) BGC from *Streptomyces* sp. MST-71458

Name	Start Position	End Position	Residues	Direction	Top Hit	Identity (%)	Coverage (%)
Orf-1	1	639	213	forward	hypothetical protein [*Streptomyces* sp. L-9-10]	77	78
DsaP	521	1213	231	reverse	DsaP [*Streptomyces scopuliridis*]	92	100
DsaO	1667	2116	150	forward	DsaO [*Streptomyces scopuliridis*]	97	100
DsaN	2290	2958	223	reverse	response regulator transcription factor [*Streptomyces* sp. L-9-10]	98	100
DsaM	2946	4157	404	reverse	DsaM [*Streptomyces scopuliridis*]	98	100
DsaL	4437	5255	273	forward	ABC transporter ATP-binding protein [*Streptomyces* sp. L-9-10]	92	99
DsaK	5261	8020	920	forward	DsaK [*Streptomyces scopuliridis*]	98	100
DsaJ	8190	9599	470	forward	DsaJ [*Streptomyces scopuliridis*]	99	100
DsaH	9830	24238	4803	forward	NRPS [*Streptomyces* sp. KCB13F003]	65	100
DsaG	24343	32280	2646	forward	DsaG [*Streptomyces scopuliridis*]	97	100
DsaF	32347	32556	70	forward	DsaF [*Streptomyces scopuliridis*]	100	100
DsaE	32662	33036	125	forward	DsaE [*Streptomyces scopuliridis*]	99	100
DsaD	33125	34258	378	forward	DsaD [*Streptomyces scopuliridis*]	97	100
DsaC	34255	34758	168	forward	DsaC [*Streptomyces scopuliridis*]	99	100
DsaB	34828	35640	271	forward	DsaB [*Streptomyces scopuliridis*]	99	100
DsaA	36620	37537	306	forward	DsaA [*Streptomyces scopuliridis*]	100	100
Orf+1	37646	38347	234	reverse	hydrolase [*Streptomyces scopuliridis*]	98	100
Orf+2	38361	38843	161	reverse	winged helix-turn-helix domain-containing protein [*Streptomyces scopuliridis*]	90	100
Orf+3	38901	40286	462	reverse	FUSC family protein [*Streptomyces scopuliridis*]	92	98

2.6 Supplementary Table 6: BLAST analysis of desotamide (*dsa*) BGC from *Streptomyces* sp. MST-94754

Name	Start Position	End Position	Residues	Direction	Top Hit	Identity (%)	Coverage (%)
Orf-1	1	639	213	forward	hypothetical protein [*Streptomyces* sp. L-9-10]	92	77
DsaP	521	1219	233	reverse	thioesterase [*Streptomyces* sp. L-9-10]	91	99
DsaO	1673	2122	150	forward	hypothetical protein [*Streptomyces* sp. L-9-10]	95	99
DsaN	2340	3008	223	reverse	response regulator transcription factor [*Streptomyces* sp. L-9-10]	100	99
DsaM	2996	4207	404	reverse	DsaM [*Streptomyces scopuliridis*]	98	99
DsaL	4494	5312	273	forward	ABC transporter ATP-binding protein [*Streptomyces* sp. L-9-10]	99	99
DsaK	5318	8086	923	forward	ABC transporter permease [*Streptomyces* sp. L-9-10]	96	99
DsaJ	8405	9814	470	forward	serine hydrolase [*Streptomyces* sp. L-9-10]	96	99
DsaI	10049	14797	1583	forward	amino acid adenylation domain-containing protein [*Streptomyces* sp. L-9-10]	90	84
DsaH	14854	24138	3095	forward	DsaH [*Streptomyces scopuliridis*]	89	99
DsaG	24246	32198	2651	forward	DsaG [*Streptomyces scopuliridis*]	89	99
DsaF	32238	32447	70	forward	MbtH family protein [*Streptomyces* sp. L-9-10]	96	98
DsaE	32544	32918	125	forward	nuclear transport factor 2 family protein [*Streptomyces* sp. L-9-10]	97	99
DsaD	32976	34133	386	forward	branched-chain amino acid aminotransferase [*Streptomyces* sp. L-9-10]	93	99
DsaC	34130	34633	168	forward	DsaC [*Streptomyces scopuliridis*]	94	99
DsaB	34705	35520	272	forward	indole-3-glycerol phosphate synthase TrpC [*Streptomyces* sp. L-9-10]	93	92
DsaR	35577	36182	202	reverse	TetR family transcriptional regulator [*Streptomyces* sp. NEAU-C40]	85	94
DsaS	36314	37213	300	forward	SDR family oxidoreductase [*Streptomyces* sp. NEAU-C40]	88	99
DsaA	38559	39476	306	forward	DsaA [*Streptomyces scopuliridis*]	93	99
Orf+1	39669	40370	234	reverse	HAD family phosphatase [*Streptomyces* sp. L-9-10]	97	99
Orf+2	40384	40866	161	reverse	Lrp/AsnC family transcriptional regulator [*Streptomyces* sp. L-9-10]	99	99
Orf+3	40938	42365	476	reverse	hypothetical protein [*Streptomyces* sp. L-9-10]	84	99

2.7 Supplementary Table 7: BLAST analysis of desotamide (*dsa*) BGC from *Streptomyces* sp. MST-127221

Name	Start Position	End Position	Residues	Direction	Top Hit	Identity (%)	Coverage (%)
Orf-1	1	633	211	forward	hypothetical protein [*Streptomyces* sp. L-9-10]	77	93
DsaP	521	1213	231	reverse	thioesterase [*Streptomyces* sp. L-9-10]	94	99
DsaO	1666	2115	150	forward	hypothetical protein [*Streptomyces* sp. L-9-10]	96	99
DsaN	2305	2973	223	reverse	response regulator transcription factor [*Streptomyces* sp. L-9-10]	100	99
DsaM	2961	4172	404	reverse	sensor histidine kinase [*Streptomyces* sp. L-9-10]	98	99
DsaL	4459	5277	273	forward	ABC transporter ATP-binding protein [*Streptomyces* sp. L-9-10]	100	99
DsaK	5283	8051	923	forward	ABC transporter permease [*Streptomyces* sp. L-9-10]	98	99
DsaJ	8370	9779	470	forward	serine hydrolase [*Streptomyces* sp. L-9-10]	97	99
DsaH	9924	24395	4824	forward	NRPS [*Streptomyces* sp. KCB13F003]	66	99
DsaG	24509	32494	2662	forward	DsaG [*Streptomyces scopuliridis*]	89	99
DsaF	32532	32741	70	forward	MbtH family protein [*Streptomyces* sp. L-9-10]	100	98
DsaE	32802	33176	125	forward	nuclear transport factor 2 family protein [*Streptomyces* sp. L-9-10]	100	99
DsaD	33234	34400	389	forward	branched-chain amino acid aminotransferase [*Streptomyces* sp. L-9-10]	94	99
DsaC	34397	34900	168	forward	YbaK/prolyl-tRNA synthetase associated domain-containing protein [*Streptomyces* sp. L-9-10]	97	99
DsaB	34987	35793	269	forward	indole-3-glycerol phosphate synthase TrpC [*Streptomyces* sp. L-9-10]	97	99
DsaT	35821	36393	191	reverse	TetR family transcriptional regulator [*Streptomyces* sp. NEAU-C40]	82	99
DsaS	36516	37412	299	forward	SDR family oxidoreductase [*Streptomyces* sp. NEAU-C40]	88	99
DsaY	37630	38028	133	reverse	-	-	-
DsaA	38751	39668	306	forward	DsaA [*Streptomyces scopuliridis*]	94	99
Orf+1	39709	40410	234	reverse	HAD family phosphatase [*Streptomyces* sp. L-9-10]	97	99
Orf+2	40424	40906	161	reverse	Lrp/AsnC family transcriptional regulator [*Streptomyces* sp. L-9-10]	99	99
Orf+3	40964	42394	477	reverse	hypothetical protein [*Streptomyces* sp. L-9-10]	90	99

2.8 Supplementary Table 8: Adenylation Domain Specificity Codes of the Wollamide (*wol*) BGC and RmoG (Orf6595)

A Domain	235	236	239	278	299	301	322	330	331	517	Observed Substrate
1AMU	D	A	W	T	I	A	A	I	C	K	L-phenylalanine
WolI A	D	V	A	M	T	G	M	V	T	K	L-tryptophan
WolH A1	D	A	L	F	V	A	A	V	A	K	L-isoleucine
WolH A2	D	A	L	F	V	A	A	V	V	K	L-leucine
WolH A3	D	A	L	W	S	G	G	V	F	K	L-leucine
WolG2 A1	D	L	T	K	G	E	E	V	G	K	L-asparagine
WolG2 A2	S	F	S	D	L	G	F	V	D	K	L-ornithine
WolG1 A1	D	L	T	K	G	E	E	V	G	K	L-asparagine
WolG1 A2	D	I	L	Q	G	E	L	I	W	K	glycine
RmoG A	D	I	L	Q	L	G	V	I	W	K	glycine

Substrate specificity codes of each adenylation domain were identified by alignment with PheA from *Bacillus brevis* (1AMU).

2.9 Supplementary Table 9: RDP analysis of all NRPS encoded adenylation domains in the *Streptomyces* sp. MST-110588 genome

**Recombinant**	**Major parent**	**Minor parent**	**Recombinant region**	**R**	**G**	**B**	**M**	**C**	**S**	**T**
wolG1A2	***wolG2A2***	***orf6595A***	**Y116-G343**	**+**	**+**	**+**	**+**	**+**	**+**	**+**
orf1237A	Unknown	*orf1236A*	I352-I448	+	+	+	+	+	+	+
orf6593A2	*orf1236A*	Unknown	M139-N354	+	-	+	-	+	+	-
orf1237A	*orf6652A*	Unknown	K223-M241	+	+	-	+	-	-	-
orf3282A	*orf6595A*	Unknown	I357-H452	-	-	+	+	-	+	-
wolHA1	*wolHA3*	*orf1227A*	N219-K316	-	-	-	+	+	+	-
wolHA2	*wolHA3*	*orf1227A*	N219-K316	-	-	-	+	+	+	-
wolG1A1	*wolHA3*	*orf1227A*	N219-K316	-	-	-	+	+	+	-
wolG2A1	*wolHA3*	*orf1227A*	N219-K316	-	-	-	+	+	+	-
orf6593A1	*orf6652A*	Unknown	K314-R389	+	-	-	-	-	+	-
orf252A	*orf253A*	*orf6595A*	T173-L202	-	-	-	+	+	-	-
orf363A	*wolG2A2*	*orf6625*	L91-L450	-	-	-	+	+	-	-
orf1224A	*orf6593A1*	*orf289A*	K84-A332	-	-	-	+	+	-	-
orf1236A	Unknown	*orf6594*	M95-I377	-	-	-	+	-	+	-

Predicted recombinants from RDP4 for which breakpoint analysis could be applied. Recombination region refers to the approximate homologous positions in the L-phenylalanine activating domain of gramicidin synthase (1AMU). Events are sorted by the whether or not (+/-) the event was predicted by a given detection method (R: RDP[14]; G: GENECONV[15]; B: BootScan[16]; M: MaxChi[17]; C: Chimaera[18]; S: SiScan[19]; L: LARD[20]).

2.10 Supplementary Table 10: cblaster analysis of minor parent BGC from *Streptomyces* sp. MST-110588

**Organism**	**MIBIG Accession**	**Product**	**6603**	**02**	**01**	**00**	**99**	**E**	**F**	**96**	**<u>G</u>**	**H**	**I**	**92**	**J**	**K**	**L**	**M**	**87**	**86**	**6585**
Streptomyces rimosus subsp. rimosus ATCC 10970	BGC0001760	Rimosamide	0	0	0	0	0	1	1	0	1	1	1	1	1	1	1	1	0	0	0
Streptomyces canus ATCC 12647	BGC0001406	Telomycin	1	1	0	0	0	0	0	0	1	0	1	0	0	0	0	0	0	0	0
Streptomyces sp. SANK 62799 SANK 62799	BGC0000288	A-503083	0	0	0	0	0	0	0	0	0	0	0	0	0	0	0	0	1	1	1

Results from a local cblaster[21,22] search querying the genes surrounding *orf6595* against a local copy of the MIBiG[22] database (16/10/19). Genes are named according to their *rmo* homologue or position in the *Streptomyces* sp MST115088 genome where no homologue is available. The minor parent involved in the recombination event discussed in this paper, *rmoG* (*orf6595*), is underlined. Cells are shaded to reflect the number of hits against each cluster.

2.11 Supplementary Table 11: Hydroxylamine trapping assay of adenylation domain specificity

Amino Acid	WolG2A2		WolG1A2		RmoG		Recombinant	
	A545	SE	A545	SE	A545	SE	A545	SE
A	0.00	3.E-04	0.00	8.E-04	0.00	6.E-04	0.01	7.E-04
C	0.00	3.E-03	0.00	1.E-03	0.00	3.E-04	0.01	6.E-04
D	0.01	1.E-03	0.00	2.E-03	0.00	6.E-04	0.01	7.E-04
E	0.00	3.E-04	0.00	6.E-04	0.00	6.E-04	0.01	1.E-03
F	0.00	7.E-04	0.00	4.E-04	0.00	2.E-03	0.01	7.E-04
G	0.00	6.E-04	0.02	7.E-03	0.03	7.E-03	0.07	3.E-03
H	0.00	1.E-03	0.00	1.E-03	0.00	1.E-03	0.01	3.E-04
I	0.00	7.E-04	0.00	5.E-04	0.00	9.E-04	0.01	3.E-04
K	0.00	3.E-04	0.00	1.E-04	0.00	1.E-03	0.01	3.E-04
L	0.00	6.E-04	0.00	6.E-04	0.00	3.E-04	0.01	9.E-04
M	0.00	3.E-04	0.00	3.E-04	0.00	3.E-04	0.01	3.E-04
N	0.01	6.E-04	0.00	2.E-03	0.00	3.E-03	0.01	6.E-04
O	0.02	2.E-03	0.00	4.E-03	0.00	7.E-04	0.01	9.E-04
P	0.00	3.E-04	0.00	1.E-04	0.00	1.E-03	0.01	0.E00
Q	0.00	1.E-03	0.00	1.E-03	0.00	2.E-03	0.01	3.E-04
R	0.00	1.E-03	0.00	5.E-04	0.00	1.E-03	0.01	6.E-04
S	0.00	9.E-04	0.00	1.E-03	0.00	3.E-04	0.01	9.E-04
T	0.00	3.E-04	0.00	1.E-04	0.00	6.E-04	0.01	7.E-04
V	0.00	2.E-03	0.00	7.E-04	0.00	9.E-04	0.01	2.E-03
W	0.00	9.E-04	0.00	2.E-04	0.00	7.E-04	0.01	1.E-03
Y	0.00	9.E-04	0.00	6.E-04	0.00	9.E-04	0.01	6.E-04

Each domain was tested against the twenty proteinogenic amino acids and L-ornithine (O). Average absorbance at 545 nm measured during the assay across at least three replicates per sample and standard errors are displayed. Absorption values are shaded according to intensity. The full dataset is available as a linked Source Data file.

2.12 Supplementary Table 12: Strains used in this study

Strain	Description	Reference
Escherichia coli ET12567/pUZ8002	Demethylating conjugative strain containing the helper plasmid pUZ8002.	[23]
Escherichia coli ET12567/pUZ8002 pBO1	Conjugation of the plasmid pBO1 to *Streptomyces* strains.	This study
Escherichia coli ET12567/pUZ8002 pBO1-*wolG2*	Conjugation of the plasmid pBO1-*wolG2* to *Streptomyces*.	This study
Escherichia coli NiCo21 (DE3)	Protein expression strain.	
Escherichia coli NiCo21 (DE3)/pET28a(+)-*orf6595*	Expression of the adenylation domain ORF6595.	This study
Escherichia coli NiCo21 (DE3)/pET28a(+)-*orf6595*; pCDFDuet-1-*wolF2*	Coexpression of the adenylation domain ORF6595 and the MbtH-like protein WolF2.	This study
Escherichia coli NiCo21 (DE3)/pET28a(+)-*wolG1A2*	Expression of the adenylation domain WolG1A2.	This study
Escherichia coli NiCo21 (DE3)/pET28a(+)-*wolG1A2*; pCDFDuet-1-*wolF2*	Coexpression of the adenylation domain WolG1A2 and the MbtH-like protein WolF2.	This study
Escherichia coli NiCo21 (DE3)/pET28a(+)-*wolG2A2*	Expression of the adenylation domain WolG2A2.	This study
Escherichia coli NiCo21 (DE3)/pET28a(+)-*wolG2A2*; pCDFDuet-1-*wolF2*	Coexpression of the adenylation domain WolG2A2 and the MbtH-like protein WolF2.	This study
Escherichia coli NiCo21 (DE3)/pET28a(+)-*wolG2MP*	Expression of the adenylation domain WolG2A2::RmoG.	This study
Escherichia coli NiCo21 (DE3)/pET28a(+)-*wolG2MP*; pCDFDuet-1-*wolF2*	Coexpression of the hybrid adenylation domain WolG2A2::RmoG and the MbtH-like protein WolF2.	This study
Escherichia coli DH5α	Cloning and propogation of vectors.	[24]
Escherichia coli DH10B	F_ mcrA (mrr-hsdRMS-mcrBC), 80lacZΔ, M15, ΔlacX74 recA1 endA1 araD 139 Δ(ara, leu)7697 galU galK λrpsL (Strr) nupG	[24]
Saccharomyces cerevisiae CEN.PK 2-1C	Assembly and propogation of pBO1 and its derrivatives.	[25]
Streptomyces sp. MST-110588	Desotamide and wollamide producing strain.	This study
Streptomyces sp. MST-127221	Desotamide producing strain.	This study
Streptomyces sp. MST-70754	Desotamide producing strain.	This study
Streptomyces sp. MST-70754/pBO1	Desotamide producing strain containing empty pBO1 (control).	This study
Streptomyces sp. MST-70754/pBO1-*wolG2*	Desotamide producing strain engineered to produce wollamides through over expression of *wolG2*	This study
Streptomyces sp. MST-71321	Desotamide producing strain.	This study
Streptomyces sp. MST-71458	Desotamide producing strain.	This study
Streptomyces sp. MST-94754	Desotamide producing strain.	This study

2.13 Supplementary Table 13: Plasmids used in this study

Plasmid	Description	Reference
pGP9	Integrative (φBT1) *Streptomyces* expression vector; propagates in E. coli for cloning/conjugation; Apr^R	[5]
pFF62A	Yeast shuttle vector used in the construction of pBO1	[6]
pUZ8002	Non-transmissible oriT mobilizing plasmid; Cm^R	[23]
pBO1	Integrative (φBT1) *Streptomyces* expression vector; propagates in *E. coli* and *S. cerevisiae* for cloning and conjugation; Apr^R	This study.
pBO1-*wolG2*	Integrative (φBT1) *Streptomyces* expression vector with *wolG2* cloned between the NdeI sites; propagates in *E. coli* and *S. cerevisiae* for cloning and conjugation; Apr^R	This study.
pET28a	*E. coli* expression vector; Kan^R	EMD Biosciences
pET28a-wolG1A1	*E. coli* expression vector with adenylation domain coding region (WolG2A1) cloned between NdeI and XhoI sites; Kan^R	This study.
pET28a-wolG2A2	*E. coli* expression vector with adenylation domain coding region (WolG2A2) cloned between NdeI and XhoI sites; Kan^R	This study.
pET28a-wolG2MP	*E. coli* expression vector with hybrid adenylation domain coding region (WolG2A2::RmoG). This vector is a derivative of pET28a-wolG2A2.	This study
pCDFDuet	*E. coli* expression vector; Sm^R	Novagen (EMD Millipore)
pcDFDuet-*wolF2*	E. coli expression vector with *wolF* cloned between NdeI and KpnI sites; Sm^R	This study.

2.14 Supplementary Table 14: Primers used in this study

Primer	Sequence	Annealing Temperature (°C)	Description
pGP9_seq_F1	gagcggcggtcgaagggagatg	50	Sequencing and colony PCR of pBO1 inserts
pGP9_seq_R1	cgagcgttctgaacaaatccag		
pBO1_pGP9_F1	aatttattcatatcaggattatcaataccatatttttgaaaaagccgtttctgtaa tgaaggagaaaactcaccgaggcacttcctcgctcactgactcg	70	Amplification of pGP9 for the assembly of pBO1
pBO1_pGP9_R1	agcagcaccatatgatcacgtttttcattcggatctttaaacagtgcgctctga tagctcagataatgattatccggcagcagaaccggaccatcaccaacgcgt tggccgattcattaa		
pBO1_pFF_F1	agaacgctcggttgccgccgggcgttttttattggtgagaatccaagctaga aatctgcattaatgaatcggccaacgcgttggtgatggtccggttctg	70	Amplification of pFF for the assembly of pBO1
pBO1_pFF_R1	ccgtattaccgcctttgagtgagctgataccgctcgccgcagccgaacgac cgagcgcagcgagtcagtgagcgaggaagtgcctcggtgagttttctcc		
pBO1_wolG2_F1	ttcgagcctccttcgagccacggggccgacgatgacgacgaccaccgga cgaacgcatcgattaattaaggaggacacatatggacgcggcggcatcac	70	Cloning of *wolG2* into pBO1
pBO1_wolG2_R1			
pCDF_wolF2_F1	aagtataagaaggagatatacatatgtcgaacccgttcg	60	Coloning of *wolF2* into pCDFDuet-1
pCDF_wolF2_R1	ctcgagtctggtaaagaaacggtaccgtgtgtacgagtggtg		
pET_wolG1_F1	catcaccacagccaggatccgaatccgacgtacgcgcagctcaacga	62	Cloning of wolG1A2 into pET28a(+)
pET_wolG1_R1	cttaagcattatgcggccgcaagcttttagatcacgcacgcctgggcca		
pET_wolG2_F1	catcaccacagccaggatccgaatccgaccttcgcggagctggaggc	62	Cloning of wolG2A2 into pET28a(+)
pET_wolG2_R1	cttaagcattatgcggccgcaagcttttacaccacggcctgcgccacgt		
pET_6595_F1	catcaccacagccaggatccgaattcgacctacgccgaactggaagc	60	Cloning of orf6595A into pET28a(+)
pET_6595_R1	cttaagcattatgcggccgcaagcttttaggcggccagctccacaccct		
pET-G2MP-1	ggccaccgacaccaccgggc	20	Cloning of wolG2A2::RmoG into pET-wolG2
pET-G2MP-2	ccagccccagcagttcggcg	20	Cloning of wolG2A2::RmoG into pET-wolG2
pET-G2MP-3	gcccggtggtgtcggtggcc	20	Cloning of wolG2A2::RmoG into pET-wolG2
pET-G2MP-4	cgccgaactgctggggctgg	20	Cloning of wolG2A2::RmoG into pET-wolG2

3. Supplementary References

1. Song, Y. *et al.* Cyclic Hexapeptides from the Deep South China Sea-Derived *Streptomyces scopuliridis* SCSIO ZJ46 Active Against Pathogenic Gram-Positive Bacteria. *Journal of Natural Products* **77**, 1937–1941 (2014).

2. Kaweewan, I., Komaki, H., Hemmi, H. & Kodani, S. Isolation and Structure Determination of New Antibacterial Peptide Curacomycin Based on Genome Mining. *Asian Journal of Organic Chemistry* **6**, 1838–1844 (2017).

3. Son, S. *et al.* Genomics-Driven Discovery of Chlorinated Cyclic Hexapeptides Ulleungmycins A and B from a Streptomyces Species. *J Nat Prod* **80**, 3025–3031 (2017).

4. Gilchrist, C. L. M. & Chooi, Y.-H. clinker & clustermap.js: automatic generation of gene cluster comparison figures. *Bioinformatics* **37**, 2473–2475 (2021).

5. Kuščer, E. *et al.* Roles of rapH and rapG in Positive Regulation of Rapamycin Biosynthesis in Streptomyces hygroscopicus. *Journal of Bacteriology* **189**, 4756–4763 (2007).

6. Schimming, O., Fleischhacker, F., Nollmann, F. I. & Bode, H. B. Yeast Homologous Recombination Cloning Leading to the Novel Peptides Ambactin and Xenolindicin. *ChemBioChem* **15**, 1290–1294 (2014).

7. Watzel, J., Hacker, C., Duchardt-Ferner, E., Bode, H. B. & Wöhnert, J. A New Docking Domain Type in the Peptide-Antimicrobial-Xenorhabdus Peptide Producing Nonribosomal Peptide Synthetase from Xenorhabdus bovienii. *ACS Chemical Biology* **15**, 982–989 (2020).

8. Price, M. N., Dehal, P. S. & Arkin, A. P. FastTree 2 - Approximately maximum-likelihood trees for large alignments. *PLoS ONE* **5**, (2010).

9. Larkin, M. A. *et al.* Clustal W and Clustal X version 2.0. *Bioinformatics* **23**, 2947–2948 (2007).

10. Ziemert, N. *et al.* The natural product domain seeker NaPDoS: A phylogeny based bioinformatic tool to classify secondary metabolite gene diversity. *PLoS ONE* **7**, (2012).

11. Conti, E., Stachelhaus, T., Marahiel, M. A. & Brick, P. Structural basis for the activation of phenylalanine in the non-ribosomal biosynthesis of gramicidin S. *EMBO J* **16**, 4174–83 (1997).

12. Kelley, L. A., Mezulis, S., Yates, C. M., Wass, M. N. & Sternberg, M. J. E. The Phyre2 web portal for protein modeling, prediction and analysis. *Nature Protocols* **10**, 845–858 (2015).

13. Tanovic, A., Samel, S. A., Essen, L.-O. & Marahiel, M. A. Crystal Structure of the Termination Module of a Nonribosomal Peptide Synthetase. *Science (1979)* **321**, 659–663 (2008).

14. Martin, D. & Rybicki, E. RDP: detection of recombination amongst aligned sequences. *Bioinformatics* **16**, 562–3 (2000).

15. Padidam, M., Sawyer, S. & Fauquet, C. M. Possible Emergence of New Geminiviruses by Frequent Recombination. *Virology* **265**, 218–225 (1999).

16. Salminen, M. O., Carr, J. K., Burke, D. S. & Mccutchan, F. E. Identification of Breakpoints in Intergenotypic Recombinants of HIV Type 1 by Bootscanning. *AIDS Research and Human Retroviruses* **11**, 1423–1425 (1995).

17. Smith, J. M. Analyzing the mosaic structure of genes. *J Mol Evol* **34**, 126–9 (1992).

18. Posada, D. & Crandall, K. A. Evaluation of methods for detecting recombination from DNA sequences: computer simulations. *Proc Natl Acad Sci U S A* **98**, 13757–62 (2001).

19. Gibbs, M. J., Armstrong, J. S. & Gibbs, A. J. Sister-scanning: a Monte Carlo procedure for assessing signals in recombinant sequences. *Bioinformatics* **16**, 573–82 (2000).

20. Holmes, E. C., Worobey, M. & Rambaut, A. Phylogenetic evidence for recombination in dengue virus. *Molecular Biology and Evolution* **16**, 405–409 (1999).

21. Gilchrist, C. L. M. *et al.* cblaster: a remote search tool for rapid identification and visualisation of homologous gene clusters. *Bioinformatics Advances* **1**, 1–10 (2021).

22. Medema, M. H. *et al.* Minimum Information about a Biosynthetic Gene cluster. *Nature Chemical Biology* **11**, 625–631 (2015).

23. Paget, M. S., Chamberlin, L., Atrih, A., Foster, S. J. & Buttner, M. J. Evidence that the extracytoplasmic function sigma factor sigmaE is required for normal cell wall structure in Streptomyces coelicolor A3(2). *J Bacteriol* **181**, 204–11 (1999).

24. Hanahan, D. *DNA Cloning: A Practical Approach*. (IRL Press, 1985).

25. Nijkamp, J. F. *et al.* De novo sequencing, assembly and analysis of the genome of the laboratory strain Saccharomyces cerevisiae CEN.PK113-7D, a model for modern industrial biotechnology. *Microbial Cell Factories* **11**, (2012).
