# Supplementary material for: Bifurcation drives the evolution of assembly-line biosynthesis
Source: Nat Commun. 2022 Jun 17;13:3498. doi: 10.1038/s41467-022-30950-z (PMC9205934; doi:10.1038/s41467-022-30950-z)
Supplement: Supplementary file 3 — Reporting Summary [file 41467_2022_30950_MOESM3_ESM.pdf]

nature portfolio

Corresponding author(s): Barrie Wilkinson (NCOMMS-21-21839B)

Last updated by author(s): April 3rd 2022

Reporting Summary

Nature Portfolio wishes to improve the reproducibility of the work that we publish. This form provides structure for consistency and transparency in reporting. For further information on Nature Portfolio policies, see our Editorial Policies and the Editorial Policy Checklist.

Statistics

For all statistical analyses, confirm that the following items are present in the figure legend, table legend, main text, or Methods section.

n/a	Confirmed	
☐	☒	The exact sample size (*n*) for each experimental group/condition, given as a discrete number and unit of measurement
☐	☒	A statement on whether measurements were taken from distinct samples or whether the same sample was measured repeatedly
☒	☐	The statistical test(s) used AND whether they are one- or two-sided<br>*Only common tests should be described solely by name; describe more complex techniques in the Methods section.*
☒	☐	A description of all covariates tested
☒	☐	A description of any assumptions or corrections, such as tests of normality and adjustment for multiple comparisons
☐	☒	A full description of the statistical parameters including central tendency (e.g. means) or other basic estimates (e.g. regression coefficient) AND variation (e.g. standard deviation) or associated estimates of uncertainty (e.g. confidence intervals)
☒	☐	For null hypothesis testing, the test statistic (e.g. *F*, *t*, *r*) with confidence intervals, effect sizes, degrees of freedom and *P* value noted<br>*Give P values as exact values whenever suitable.*
☒	☐	For Bayesian analysis, information on the choice of priors and Markov chain Monte Carlo settings
☒	☐	For hierarchical and complex designs, identification of the appropriate level for tests and full reporting of outcomes
☒	☐	Estimates of effect sizes (e.g. Cohen's *d*, Pearson's *r*), indicating how they were calculated

Our web collection on statistics for biologists contains articles on many of the points above.

Software and code

Policy information about availability of computer code

Data collection	Thermo Scientific Xcalibur 4.3, RDP4
Data analysis	HGAP2.0 , antiSMASH v4.0 and 5.0, NRPSsp, NRPSpredictor2, BLASTp, PyMOL2.5, RDP4, FastTree 2, ClustalW, Molecular Operating Environment (MOE) 2019.0102, cblaster, clinker, Prodigal, AutoMLST, Phyre2, Thermo Scientific Freestyle 1.6

For manuscripts utilizing custom algorithms or software that are central to the research but not yet described in published literature, software must be made available to editors and reviewers. We strongly encourage code deposition in a community repository (e.g. GitHub). See the Nature Portfolio guidelines for submitting code & software for further information.

Data

Policy information about availability of data

All manuscripts must include a data availability statement. This statement should provide the following information, where applicable:

- Accession codes, unique identifiers, or web links for publicly available datasets
- A description of any restrictions on data availability
- For clinical datasets or third party data, please ensure that the statement adheres to our policy

Data supporting the findings of this work are available within the paper and its Supplementary Information file, or in publicly available databases. The DNA sequence data for the genome assembly and biosynthetic gene clusters (BGCs) generated in this study have been deposited in GenBank. The genome sequence of Streptomyces sp. MST-110588 has been deposited under the accession code GenBank: CP074380 [https://www.ncbi.nlm.nih.gov/nuccore/ CP074380]. The desotamide BGCs have been deposited under the accession codes as follows: Streptomyces sp. MST-70754, GenBank: MZ093610 [https://www.ncbi.nlm.nih.gov/nuccore/MZ093610]; Streptomyces sp. MST-71458, GenBank:MZ093611 [https://www.ncbi.nlm.nih.gov/nuccore/MZ093611]; Streptomyces sp. MST-71321, GenBank: MZ093612 [https://www.ncbi.nlm.nih.gov/nuccore/MZ093612]; Streptomyces sp. MST-94754, GenBank: MZ093613 [https://www.ncbi.nlm.nih.gov/

nuccore/MZ093613]; and Streptomyces sp. MST-127221, GenBank: MZ093614 [https://www.ncbi.nlm.nih.gov/nuccore/MZ093614]. The crystal structure coordinates of the docking domain protein PaxC_NDD–PaxB_CDD were retrieved from the RSCB Protein Data Bank file PDB-ID:6TRP_1[DOI: 10.221o/pdb6trp/pdb]. Biochemical data for analysis of adenylation domain substrate specificity is available as a Source Data file. A reporting summary for this Article is available as a Supplementary Information file.

Field-specific reporting

Please select the one below that is the best fit for your research. If you are not sure, read the appropriate sections before making your selection.

- [x] Life sciences
- [ ] Behavioural & social sciences
- [ ] Ecological, evolutionary & environmental sciences

For a reference copy of the document with all sections, see nature.com/documents/nr-reporting-summary-flat.pdf

Life sciences study design

All studies must disclose on these points even when the disclosure is negative.

Sample size	The study is based on the observation of microbial strains. Therefore, the sample is based upon the strains available to the researchers.
Data exclusions	No data was excluded.
Replication	Biochemical analysis of the adenylation domains was replicated using three independent assays for the same batch of protein (technical replicate). Prior to data generated for the recorded run, a preliminary experiment was run with a different batch of protein and in each case gave essentially the same result.<br>For the analysis of desotamide and wollamide production by Streptomyces strains, three biological replicates were run for each strain. These experiments were run twice, at different times, and on each occasion using the same methodology but by a different scientist.
Randomization	No randomization was performed. We used defined genome sequence data, and generated a defined set of mutant strains; for each mutant a number of individual isolated progeny were assayed, and all data was used. Similarly, for biochemical assays a defined number of mutant protein samples were prepared, all of which were assayed for specific activity and all data generated was utilised in the subsequent analysis.
Blinding	No blinding was performed. Experiments were designed, performed and analysed by the same individual, and the methodology did not allow for blinding.

Reporting for specific materials, systems and methods

We require information from authors about some types of materials, experimental systems and methods used in many studies. Here, indicate whether each material, system or method listed is relevant to your study. If you are not sure if a list item applies to your research, read the appropriate section before selecting a response.

Materials & experimental systems

n/a	Involved in the study	
x		Antibodies
x		Eukaryotic cell lines
x		Palaeontology and archaeology
x		Animals and other organisms
x		Human research participants
x		Clinical data
x		Dual use research of concern

Methods

n/a	Involved in the study	
x		ChIP-seq
x		Flow cytometry
x		MRI-based neuroimaging
